# Exploring the Potentials of Wearable Technologies in Managing Vestibular Hypofunction

**DOI:** 10.3390/bioengineering11070641

**Published:** 2024-06-24

**Authors:** Ameer Mohammed, Shutong Li, Xiao Liu

**Affiliations:** 1 School of Information Science and Technology, Fudan University, Shanghai 200433, China; ameer@fudan.edu.cn (A.M.); 20210720051@fudan.edu.cn (S.L.); 2 State Key Laboratory of Integrated Chips and Systems, Fudan University, Shanghai 201203, China

**Keywords:** sensory augmentation, sensory substitution, vestibular hypofunction, vestibular rehabilitation, vestibular management, wearable devices

## Abstract

The vestibular system is dedicated to gaze stabilization, postural balance, and spatial orientation; this makes vestibular function crucial for our ability to interact effectively with our environment. Vestibular hypofunction (VH) progresses over time, and it presents differently in its early and advanced stages. In the initial stages of VH, the effects of VH are mitigated using vestibular rehabilitation therapy (VRT), which can be facilitated with the aid of technology. At more advanced stages of VH, novel techniques that use wearable technologies for sensory augmentation and sensory substitution have been applied to manage VH. Despite this, the potential of assistive technologies for VH management remains underexplored over the past decades. Hence, in this review article, we present the state-of-the-art technologies for facilitating early-stage VRT and for managing advanced-stage VH. Also, challenges and strategies on how these technologies can be improved to enable long-term ambulatory and home use are presented.

## 1. Introduction

The vestibular system located inside the inner ear is responsible for balance and spatial orientation [1]. It influences gait, gaze, posture, and coordination between eye and head movements. When vestibular function is impaired or lost, it results in vestibular hypofunction (VH) [2]. This could have serious consequences on a subject’s postural control, gaze stabilization, spatial orientation, and mental state. Hypofunction could be unilateral or bilateral [3]. Unilateral hypofunction is typically due to the loss or diminished function in one of the vestibular systems, either right or left. On the other hand, bilateral hypofunction is loss or diminished function on both right and left. People with bilateral vestibular impairments in the US and Europe are approximately 500,000–700,000, with the figures reportedly downplayed due to the difficulty in diagnosis [4]. Vestibular loss is mainly common among the elderly. VH is not only a devastating condition for the elderly but also puts a significant financial burden on the healthcare system [5].

Proper health monitoring in patients with VH not only enhances VH management but also improves quality of life. Despite this, the potential of assistive technologies for VH management remains underexplored over the past decades. Vestibular loss, which progresses over time, presents differently in its early and advanced stages. Initially, symptoms are intermittent and manageable with medication or vestibular rehabilitation therapy (VRT). However, as the condition advances, symptoms become constant. VRT is administered as the first line of therapy to reinforce weak vestibular senses through habituation and adaptation enabled by other senses [6]. VRT is an exercise-based program primarily designed to reduce vertigo and dizziness, gaze instability, imbalance, and risk of falls. During VRT, wearable systems are essential for tracking disease progression outside clinical settings. This is due to the need for periodic tests to assess VRT improvements, which otherwise would necessitate frequent, inconvenient hospital visits. Moreover, clinical tests may not accurately reflect the real-life scenarios of VH. Wearable assistive technologies allow for the monitoring of sensory information to improve rehabilitation and enable a more active living for impaired persons.

At advanced stages of VH, novel techniques for sensory substitution and sensory augmentation, like vestibular implants [7], biomimetic hair cells [8], noisy Galvanic vestibular stimulation (nGVS) [9], and biofeedback devices [10], are administered. Some of these approaches are deployed to target the root cause of vestibular deficits, which are sensorineural; that is, they stem from damage to the vestibular apparatus and/or vestibular nerve. However, using invasive surgical interventions like vestibular implants and biomimetic hair cells, there is a great risk of partial or complete hearing loss [11]. To mitigate this risk, non-invasive techniques for managing VH can be adopted, such as non-invasive electrical stimulation. It is desirable to steer the stimulus current to the vestibular nerve to elicit an appropriate neural response. However, this may not be easily achieved with a non-invasive approach because of the limited control of the current spread to adjoining organs [7].

So far, wearable systems for managing VH have shown great potential in providing real-time, accurate, comprehensive, and personalized therapy. Hence, this work presents the major technologies for facilitating VRT, as well as managing VH at more advanced stages. Wearable solutions for vestibular management that could be adopted for long-term ambulatory and home use are presented, as well as strategies on how these technologies can be improved for future use. The main contributions of this work are as follows:

- It provides an overview of the management of VH using assistive technologies at different stages of VH. This work presents the first instance in which technologies used for VH management are categorized based on the stage in which they are used—early- or advanced-stage intervention. For early-stage intervention, it highlights the role of these technologies in facilitating VRT and classifies them based on their mobility and portability. Various platforms, including virtual reality (VR), PC-based, videogame-based, mobile phone-based, and tablet-based methods, are discussed.
- For advanced-stage technological interventions for VH, it explores the use of sensory augmentation and sensory substitution technologies. It discusses these using mathematical analogies and flow diagrams. To the authors’ best knowledge, this is the first time the content has been organized from an engineering perspective by using mathematical analogies and flow diagrams to show how these techniques can enhance or replace vestibular function by providing new avenues for vestibular perception.
- Based on the limitations of current wearable technologies for VH management presented in the article, it outlines the key technological advancements that would be required for VH management technologies of the future.

The rest of the manuscript is organized as follows. Section 2 provides insights on VH, with Section 3 and Section 4 discussing technological interventions for early and advanced stages of VH, respectively. The future of wearable technologies for VH is presented in Section 5, with concluding remarks presented in Section 6.

## 2. Understanding Vestibular Hypofunction

The vestibular system is dedicated to gaze stabilization, postural balance, and spatial orientation [12]. These functions are maintained using vestibular reflexes. Vestibular reflexes are initiated by signals from the vestibular system. Having a healthy vestibular system is crucial for our ability to interact with our surrounding environment. For example, for quick head movement, the vestibulo-ocular reflex (VOR) helps stabilize the gaze by making rapid eye movements in the opposite direction. Aside from VOR, other vestibular reflexes include the vestibulo-spinal reflex (VSR) [13], vestibulocollic reflex (VCR) [14], and vestibulo-cerebellum reflexes. Similar to VOR, VCR also assists in gaze stabilization, while VSR helps stabilize posture, and vestibulo-cerebellum reflexes support spatial orientation. 

**Figure 1:**
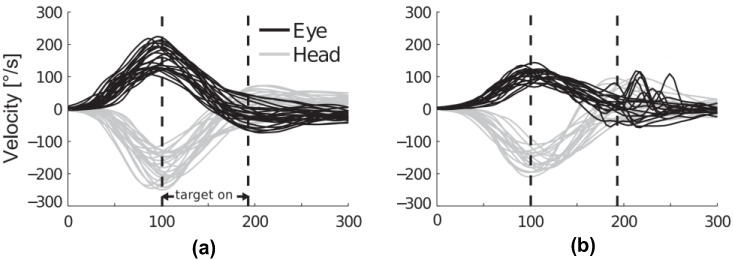
Eye and head velocity during head impulses for (**a**) normal VOR gain and (**b**) reduced VOR gain for VH patients (image from [17]).

The VOR is the fastest reflex in humans [15], making it the most difficult reflex to manage when impaired. [1,16]. The VOR gain is defined as the ratio of eye-to-head movement. It is a key measure of the matching between vestibular and visual systems. For a healthy patient, this should be around 1, indicating equal but opposite eye and head movement velocities (as shown in Figure 1a). For a VH patient, the eye velocity lags behind the head velocity (as shown in Figure 1b). This makes it a reliable metric for assessing vestibulopathy. Monitoring the VOR gain not only offers insights into the status of the vestibular system [16] but also serves as objective evidence of vestibular dysfunction [1]. Therefore, modulating the VOR could mitigate vestibular loss [16].

VH can be debilitating, affecting a person’s ability to perform daily activities and leading to a decreased quality of life. In severe cases, it can lead to falls and injuries, further exacerbating the problem [18]. VH can occur as a result of aging or due to a variety of diseases and conditions, including vestibular neuritis, vestibular schwannoma, and Meniere’s disease, among others [2,19]. Natural aging results in changes to the vestibular system because of age-related vestibular hair cell loss and neuronal cell loss in parts of the brain involved in processing vestibular information [20]. Therefore, VH represents a significant public health issue, especially for the elderly [21]. Understanding this condition and developing effective methods for its assessment is essential.

Both unilateral vestibular hypofunction (UVH) and bilateral vestibular hypofunction (BVH) are treated with VRT. VRT is an exercise-based treatment program designed to promote vestibular adaptation and substitution [22]. VRT is mostly effective for patients with UVH, where it promotes central compensation and relies on the other ear (the normal side) to perform most of the inner ear functions [3]. VRT can also provide benefits for patients with BVH; however, the complete loss of inner ear function presents significant management challenges. During VRT, regular tests are carried out to monitor improvements in vestibular function. These tests are mostly conducted within clinical settings, but wearable systems could ease this process. More details on VRT, VH, and its diagnosis are provided in the following review articles [2,23,24,25].

## 3. Technological Interventions for Early-Stage VH

At the early stages of VH, VRT is the main management strategy for patients with partial vestibular loss [3]. It involves exercises designed to enhance gaze stabilization, leveraging the adaptive potential of a hypo-functioning VOR [26]. Patients are guided to perform active head movements while maintaining visual focus on a target [15]. These conventional exercises improve the patient’s quality of life, even though they do not address the root causes of the deficiency.

The vestibular, somatosensory, and visual systems each play unique roles in stabilizing gaze and controlling posture and orientation [27,28,29]. The brain assigns different importance to each system based on the frequency of movements, and this varies from person to person, necessitating ongoing learning and adjustment [30]. VRT results in the brain learning to use other senses (visual and somatosensory) to compensate for the deficient vestibular system, and it achieves this through sensory adaptation and substitution [6]. For most patients with VH, the amount of restoration of vestibular function via VRT is very limited [31]. 

**Figure 2:**
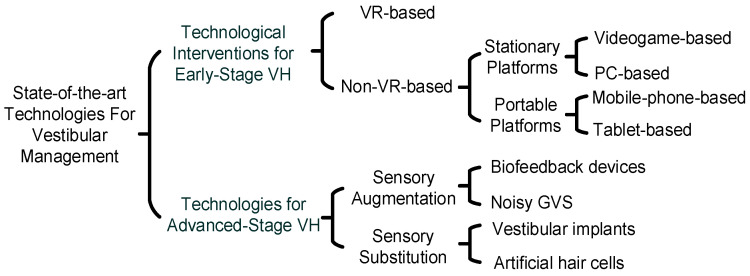
Summarizing the various wearable technologies for vestibular management.

Currently, there are studies on the use of different types of technological devices for the rehabilitation and management of BVH [3]. The choice of technology and its usefulness depend on the individual patient’s symptoms, disease stage, needs, and goals. Vestibular management includes medication, lifestyle changes, surgery, and technologies. Our focus is on the use of technologies for VH management; these are broadly divided into two categories: (i) VRT technologies at the early stages of VH and (ii) technologies for sensory augmentation or substitution at the advanced stages of VH. Figure 2 summarizes the various wearable technologies reported in the literature.

### 3.1. VR-Based Methods

Virtual reality (VR) systems specific to vestibular rehabilitation have been developed to achieve three primary goals: to reduce symptoms (vertigo, dizziness, and tolerance to visual motion), to improve gaze stabilization, and to improve postural stability [32]. VR can provide amazing benefits for a person with a vestibular disorder. For instance, immersive VR games have been demonstrated to improve the VOR response and balance [33]. In addition, VR can simulate real-world situations that might be difficult for patients to encounter in a controlled clinical setting. This allows patients to practice handling these situations in a safe environment, which can help improve their confidence and reduce anxiety related to their symptoms.

### 3.2. Non-VR Based Methods

These are subdivided based on the mobility and portability of the rehabilitation platforms. These include stationary and portable platforms, which are discussed below.

#### 3.2.1. Stationary Platforms

These include PC and videogame-based systems for VRT. These are discussed below.

**PC-Based Systems:** Different computer-aided systems have been created to help stabilize gaze. One such system uses the interactive Wiimote for exercises [34]. It employs infrared LEDs and a receiver to assist patients with balance issues. The system measures the patient’s head movements, enabling real-time tracking of their progress on the rehabilitation platform. 

**Videogame-Based Systems:** Regarding the application of videogame-based systems for gaze stabilization exercise, an interactive videogame has been used for gaze stabilization exercise in children [35]. The system incorporated real-time head-motion detection, such that an angular acceleration threshold can be set to increase the exercise’s difficulty level in real-time. In another study, the efficacy of commercially available videogames for vestibular rehabilitation was investigated [36]. It was found that some of the video games produced optokinetic stimulation that presented similar effects as both habituation and gaze stabilization exercises. This makes them a useful assistive technology for facilitating VRT.

#### 3.2.2. Portable Platforms

These include mobile phones and tablet-based systems for VRT. These are discussed below.

**Mobile Applications:** There are several mobile applications designed to assist with vestibular rehabilitation. During the COVID-19 pandemic, due to the restrictions on visiting clinics in person, the use of smartphones in diagnosing VH was explored [37]. This approach allows for remote consultation and management of patients with suspected diagnosis of VH. Innovative app-based games have been developed to boost exercise performance for patients with VH [38]. These apps often include a variety of exercises that patients can do at home, and they may also provide educational resources to help patients better understand their condition. These exercises use an inertial measurement unit (IMU) to control a character on the screen, responding to the participants’ physical movements [38]. Some apps even allow patients to track their symptoms and progress, which can be shared with their healthcare providers to enable them to adjust patients’ VRT.

**Tablet-Based:** An example of this is VestAid, which is a tablet-based technology for objective exercise monitoring during VRT [39]. It uses the tablet’s camera to automatically assess patient performance and compliance with exercise parameters. The system provides physical therapists with updates on head speed, gaze fixation compliance, and perceived difficulty (pre- and post-exercise symptoms) metrics through a web-based portal. Another tablet-based system is StableEyes [15]. StableEyes implements unassisted exercises that can be performed at home by patients with VOR hypofunction. The device consists of a base unit and a head unit. The latter contains inertial sensors for measuring the instantaneous 3D orientation of the head in space and an integrated circuit to dynamically control the position of a laser target in space with respect to head movements. This approach relies on the patient tracking a visual target that moves as a function of the head motion but at a different speed so that the VOR is challenged to increase through adaptation in order to stabilize the retinal image of the target.

## 4. Advanced-Stage Intervention Technologies: Sensory Augmentation and Sensory Substitution

**Figure 3:**
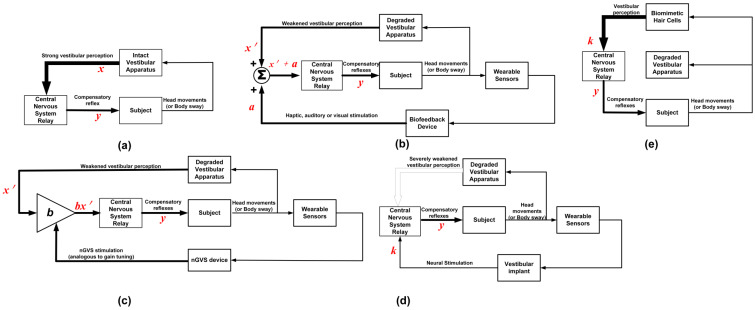
Flow diagram for vestibular perception. (**a**) Normal subjects with strong vestibular perception (broad feedback arrow). (**b**) Sensory augmentation using biofeedback devices showing weakened vestibular perception (narrow feedback arrow). (**c**) Sensory augmentation using nGVS showing weakened vestibular perception (narrow feedback arrow). (**d**) Sensory substitution using vestibular implant showing severely weakened vestibular perception (unshaded feedback arrow). (**e**) Sensory substitution using biomimetic hair cells for generating vestibular perception (broad feedback arrow).

Several technical approaches for managing BVH have been proposed [40]. Figure 3 summarizes the various methods of vestibular perception. Figure 3a shows the vestibular perception of normal patients, while Figure 3b–e. show how different technological interventions can be used to provide new avenues for vestibular perception. In normal vestibular perception, shown in Figure 3a, head movements are accompanied by strong compensatory reflexes. A mathematical analogy for this is

(1) y=f(x)

where the input, *x*, is analogous to the strong vestibular perception, and the output, *y*, is analogous to the compensatory reflex. Figure 3b,c shows how sensory augmentation can be used to enhance weakened vestibular perception. In both configurations, wearable sensors are used to provide real-time feedback about a patient’s balance and movement. Figure 3d,e uses neural stimulation and biomimetic hair cells, respectively, to trigger compensatory reflexes due to vestibular perception.

### 4.1. Sensory Augmentation

In Figure 3b,c, sensory augmentation is used to enhance weakened vestibular perception. In both configurations, wearable sensors are used to provide real-time feedback about a patient’s balance and movement. A bio-feedback device achieves sensory augmentation by stimulating other senses in response to changes in movement or posture to reinforce weakened vestibular sensation (shown in Figure 3b). The mathematical analogy is

(2) $$y=f(x^{\prime }+a)$$

where the input, *x*′, is analogous to weakened vestibular perception; the other input, *a*, is the reinforcement signal (which could be via haptic, visual, or auditory feedback); and the output, *y*, is analogous to the compensatory vestibular reflex. The reinforcement signal, *a*, is used to augment the vestibular perception *x*′.

The process of sensory augmentation using nGVS is shown in Figure 3c. The mathematical analogy is

(3) $$y=\;f(b\cdot x^{\prime })$$

where *x*′ is analogous to weakened vestibular perception, *b* is analogous to the gain factor as a result of the nGVS stimulation, and *y* is analogous to the compensatory vestibular reflex. Sensory augmentation is suitable for patients with partial BVH, which involves enhancing the remaining senses to compensate for vestibular loss [41]. The following section provides more detail on sensory augmentation using biofeedback devices and nGVS.

#### 4.1.1. Biofeedback Devices

Biofeedback devices enhance missing feedback from a defective sensory system with cues from other sensory sources (as shown in Figure 3b). For VH management, biofeedback devices have shown promise [3]. To use biofeedback devices for sensory augmentation, patients are fitted with inertial sensors that transduce information related to body motion to trigger balance-related feedback cues either by vibrotactile stimulation of the torso, auditory stimulation, or electro-tactile stimulation on the tongue [40]. Sensory bio-feedback systems are usually composed of the following [42]:

- A balance assessment sensor;
- An algorithm extracting meaningful postural information;
- An actuation device that provides relevant information to the subject in relation to the activity. This actuation signal may take the form of visual, auditory, or vibrotactile cues [10].

Using either vibrotactile or auditory feedback, these devices can be used to enhance balance and postural stability in patients. Studies have shown how cues have been used to make postural corrections or stabilize the posture of BVH patients [43]. In [44], a biofeedback device (a balance belt) was developed that aimed to reduce postural imbalance in patients by providing haptic stimulation whenever the patient’s body swayed beyond a desired threshold. The device demonstrated the potential to non-invasively improve the postural balance of patients with bilateral vestibulopathy. Wearable biofeedback devices can provide objective, real-time data about biomechanical or physiological body parameters [45]. The data from these devices can be used to track patient progress over time. This can help healthcare providers adjust treatment plans and provide patients with real-time feedback on improvement, thus enabling patients to adjust their movements, gait, and postures to achieve better outcomes. 

In addition, studies have shown that the real-time use of visual, vibrotactile, auditory, and multimodal sensory augmentation technologies can improve balance during static and dynamic activities within a laboratory setting [10]. Also, attempts have been made to enhance vestibular function using vibrotactile feedback, whereby deviations in patient posture led to the delivery of vibrotactile stimulation in response to changes in verticality [46]. In other cases, acoustic signals were used [47]

Results from the literature have shown that vibrotactile feedback of body motion reduces the number of falls in people with severe VH during standard clinical tests when there were no applied external perturbations. Nonetheless, the location of the actuation device affects the response time of the system, with better response time for head-located vibrotactile stimuli compared to the torso and sternum [48]. Aside from vibrotactile feedback, visual feedback has been explored for sensory enhancement. Inputs from the visual system are used together with inputs from the vestibular and somatosensory systems to maintain balance [10]. This is because the visual system provides the central nervous system with information about the direction of verticality. Hence, people with vestibular loss can be expected to have increased reliance on the visual and somatosensory systems for spatial orientation and mainly rely on somatosensory inputs when their eyes are closed [10].

In [49], which investigated the effect of feedback modality on balance performance during standing tasks, it was observed that subjects with vestibular loss performed better while using visual feedback compared to vibrotactile feedback. This was because, unlike vibrotactile feedback, visual feedback provided information about current and future positions [49]. The challenges of using auditory or visual feedback are that patients with vestibular deficits depend strongly on vision and hearing for communication and interaction with the environment, even more than healthy persons. Using the visual and auditory input to replace vestibular function will cause it to interfere with the basic function of these senses [30].

When using a biofeedback device, the external feedback provides the user with information about the nature of movements [50]. In addition, patients who have reached a plateau in their vestibular therapy programs can use sensory augmentation to further improve functional recovery [51]. Feedback platforms can be designed that provide patient-specific management based on the feedback signals patients are most attuned with—visual, auditory, or vibrotactile. While performing VRT, biofeedback devices have been shown to reduce body sway in real time. However, it is not clear if these have better effects on patients than doing the VRT alone [52,53]. On the other hand, other studies have reported significant improvements in the use of biofeedback devices to augment VRT [54,55]. Depending on how much cognition is required to utilize feedback, biofeedback devices may not be practical for persons who have a high risk of falling [10]. Biofeedback devices appear to be most useful during quiet and dynamic standing and during the recovery phases of mild to moderate surface perturbations. Nevertheless, their usefulness during locomotor activities and transitions between seated and upright stances appears to be limited [10]. Biofeedback devices can be used to reduce postural sway instantaneously. However, for gait and other complex movements, a more sophisticated augmentation system may be required, like the nGVS. It will be worth exploring if nGVS could be adopted for long-term use.

#### 4.1.2. Noisy GVS 

**Figure 4:**
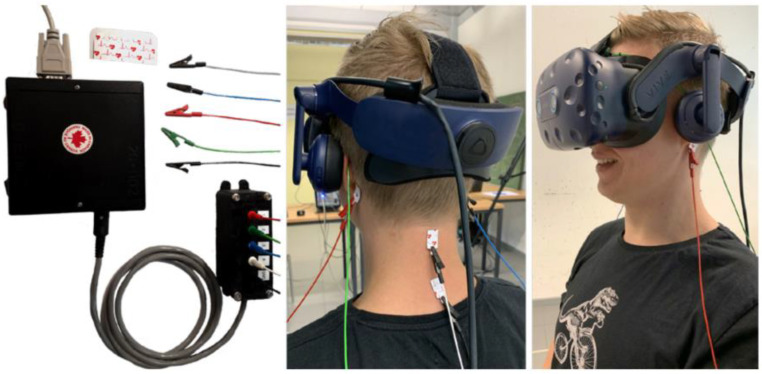
GVS with a VR headset for managing cybersickness. Stimulation electrodes are on the right and left mastoid, while ground and neutral electrodes are on the nape (image from [57]).

Galvanic vestibular stimulation (GVS) is a non-invasive method of electrically stimulating the vestibular system [56]. It involves applying alternating currents at different frequencies and amplitudes through electrodes on the mastoid bones (shown in Figure 4). The purpose of GVS is to stimulate the vestibular system in a controlled manner for various applications, ranging from medical treatments to enhancing VR experiences. It has been used to help reduce cybersickness by reinforcing absent vestibular cues while using VR [57]. Also, supra-threshold GVS can evoke a sensation of body rotation and affect verticality perception [58]. Attempts have been made to improve and optimize residual vestibular function in BVH patients using nGVS. 

Noisy GVS works on the principle of stochastic resonance, wherein the signal processing in sensory systems can be improved by adding an appropriate level of noise to the system [40]. Noisy GVS has unique impacts on the vestibular system [59], thereby influencing vestibular function differently. The added noise is not random; however, it is a carefully controlled input that can actually enhance sensory signals by lowering the vestibular threshold [3,60]. In [61], it was reported that nGVS resulted in a noise-induced lowering of the vestibulospinal threshold, which enabled subjects to sense and process usually unrecognized occurring subthreshold vestibular signals. This resulted in improved balance control in healthy subjects and patients with VH. Noisy GVS is a promising technique for the management and rehabilitation of VH. Noisy GVS plays an important role in understanding sensory signal processing in the vestibular system under normal and pathological conditions [62]. It can affect eye movement and body sway. It can generate eye movements in the absence of head/body motion [63]. In a recent study, it was found that it modulates the body sway and muscle activity of the lower limbs [64]. As such, it can affect both eye movement and body sway, making it useful for diagnosing and managing VH.

Noisy GVS treatment involves administering stimulation at intensities ranging from 0 to 0.7 mA, which is well tolerated and does not cause disequilibrium in patients [9]. Studies have shown that nGVS can improve body balance in patients with bilateral vestibulopathy for several hours after the end of the stimulus [65]. It has been shown to partially restore vestibular function and stabilize stance and gait in patients with incomplete bilateral vestibulopathy [66]. Noisy GVS can be combined with vestibular rehabilitation for the treatment of unilateral vestibulopathy [67]. This combination can accelerate static and dynamic vestibular compensation after unilateral vestibulopathy. However, when combined with VRT in patients with BVH during treatment sessions, nGVS did not induce synergistic treatment effects. Hence, rather than being applied in parallel, nGVS and VRT might be complementary therapeutic options with nGVS, being used during postural activities in daily life like walking. It has also been demonstrated that by using optimal nGVS, postural stability was considerably improved in 90% of patients [40]. In the study, compared to sham stimulation, stimulation at optimal nGVS reduced body sway velocity by 25 ± 14%, the root mean square of body sway by 22 ± 18%, and body sway area by 32 ± 26%. 

In another study [57], omnidirectional GVS was used to mitigate cybersickness in VR applications. One of the most accepted theories indicates that cybersickness is caused by the visually induced impression of ego-motion while physically remaining at rest [57]. Their hypothesis seems to be that cybersickness is caused by overcompensation for certain angles. Compensation could be caused by the low sensitivity of the receptor hair cells at those angles. These angles could be complemented with non-invasive vestibular stimulation through nGVS. The researchers believe GVS restores the oculo-vestibular recoupling, thereby complementing eye motion. Moreover, Parkinson’s disease patients, who often suffer from VH [68] and related movement, balance, and gait issues, have been found to benefit from nGVS [69]. This treatment has also shown promise for stroke patients [70]. Essentially, nGVS can alleviate impairments related to standing, balance, and gait. Also, in the context of space travel, astronauts experience sensory conflicts due to inadequate otolith stimulation in microgravity. This requires a sensory re-weighting process emphasizing vision and proprioception. Upon returning to Earth’s gravity-rich environment, astronauts must readjust, a process that can take days or weeks [70]. Wearable nGVS devices could potentially expedite this adaptation process. 

During nGVS, administering stimulation at high current intensities may cause undesired spasms due to the dispersion of electric current to the facial nerve [71]. Also, another challenge of nGVS is that a sweet spot for the additive noise needs to be found such that the stimulation is efficacious. Nonetheless, nGVS is considered a useful strategy for balance rehabilitation since it has the effect of lowering the perception threshold for VH patients [72]. For nGVS, the main challenges that may need addressing include improving stimulation focus, determining the optimal stimulation parameters, identifying the optimal current paths that induce body sway and eye movements, and the type of noise that may be required for each symptom. In addition, it will be necessary to determine how long potential benefits last [70]. 

So far, nGVS shows great promise as an assistive technology for those with a variety of neurological impairments or older adults at risk of falling. However, it is unclear whether BVH symptoms like impaired gaze stabilization (mainly oscillopsia) and spatial orientation could benefit from nGVS treatment [40]. Also, nGVS is less targeted than vestibular implants because nGVS requires the use of surface electrodes [30]. While both invasive and non-invasive electrical stimulation of the vestibular system have proven beneficial for patients, the optimal choice in terms of practicality and efficiency remains undetermined. Table 1 summarizes some state-of-the-art studies that implemented nGVS for managing vestibular-related symptoms.

### 4.2. Sensory Substitution

There is no medical treatment to restore lost vestibular function, and physical therapy is only mildly effective for BVH patients [73]. Nonetheless, sensory substitution using vestibular implants has the potential to rehabilitate balance-impaired patients and partially restore vestibular function [30,74]. The concept of vestibular implants is based on the technology and principles of the cochlear implant [75]. Different vestibular implant prototypes are currently being evaluated regarding feasibility and functionality. So far, all of them have shown promise in activating different types of vestibular reflexes. In addition to vestibular implants, research in sensory substitution using biomimetic hair cells is ongoing.

Vestibular implants and biomimetic hair cells involve sensory substitution as they provide alternative means to replace the missing sensory input via an artificial channel. Due to their invasive nature, they are more suitable for patients with complete BVH, while sensory augmentation is used for patients with partial BVH.

#### 4.2.1. Vestibular Implants

An emerging area of vestibular research is looking into surgically implanted vestibular prostheses, which generate electrical signals based on head motion that are delivered to the ampullary nerves [76]. Vestibular implants/prostheses work by replacing the lost natural afferent spike activity in the vestibular nerve with electrical stimulation [4]. Vestibular implants take advantage of MEMS gyroscopes and accelerometers to provide head movement signals to trigger the stimulators [77]. The process of which is shown in Figure 3d. The mathematical analogy is

(4) $$y=\;g\left(k\right)$$

where *k* is analogous to the artificial stimulus from the vestibular implant, and *y* is analogous to the compensatory vestibular reflex due to the stimulation signal. The stimulus, *k*, is used to replace strong vestibular perception, *x*, or the severely weakened vestibular perception, *x*′, since the subject has little or no vestibular perception. The transfer function from the artificial input, *k*, to the output, *y*, may be different from the transfer function of a healthy subject and is denoted as *g*. This stimulation smoothens and controls eye movements as well as improves balance and gaze stabilization, ultimately improving the lives and function of patients living with BVH [76]. 

Vestibular implants have been successfully demonstrated in clinical practice. Animal studies have been developed that stimulate the semicircular canals to control eye movements based on the perceived head movements [77]. This device uses multichannel sensing capable of sensing angular velocity about three orthogonal axes and asynchronously stimulating each of the three ampullary nerves, allowing partial restoration of VOR responses for head rotation about any axis. In [1], it was demonstrated that using a 3D gyroscope to track motion and motion-modulated electrical stimulation of the ampullary branches of the vestibular nerve in patients with bilateral vestibulopathy could restore major vestibular function. The system used biphasic stimulation pulses with a fixed frequency, which was modulated in amplitude around a baseline. Also, the VOR gain increased with increasing modulation amplitude. These results imply that the vestibular implant using an amplitude modulation response has the potential to become a clinically useful device for vestibular therapy [1]. Compared to the current frequency- or amplitude-based stimulation, adaptive stimulation provides more sophisticated stimulation patterns since it mimics the natural function of the vestibular system’s communication with afferent neurons. Aside from restoring vestibular function, vestibular prostheses combined with cochlear implants (both driven from the same source) have the potential to restore auditory function since many patients with VH also have hearing loss. 

The main challenge with vestibular implants is creating a device that can effectively interface with afferent neurons to restore balance [71]. This requires the implant to mimic the natural function of the vestibular system. Another issue is the hearing deficit post-implantation, which led to the evolution of bilateral implants to a unilateral device [74]. This, however, necessitated the development of specific stimulation methods for encoding bidirectional movements using a unilateral device [74]. For the unilateral device, optimizing electrical stimulation parameters, considering electrode design and location, can improve dynamic range, selectivity, and end-organ responses [77] while limiting current spread to unintended targets [78]. For vestibular implants, the surgical procedure and electrode design need refinement for precise implantation, minimal labyrinth damage, and preservation of residual vestibular and cochlear function. The use of imaging techniques and biophysiological measurements during surgery could assist in achieving these objectives. Table 2 summarizes some of the commercially available vestibular prostheses.

#### 4.2.2. Biomimetic Hair Cells

There is progress in the development of biomimetic hair cells to bypass lesioned hair cells for the electrical stimulation of the primary afferent neurons to enable the transmission of sensory signals from the vestibular system to the brain [71]. These devices are intended to replicate the transduction of the mechanical input to the electrical output of the inactive vestibular (receptor) hair cells. A flow diagram of how the biomimetic hair cells can be used as a substitute for natural vestibular transduction is shown in Figure 3e. The mathematical analogy is

(5) $$y=\;f\left(k\right)$$

where *k* is analogous to the strong vestibular perception but via the biomimetic hair cells instead of the original hair cells in the ampullary cupula of the subject, and *y* is analogous to the compensatory vestibular reflex. The technology for biomimetic hair cells is still under development and far from maturity [71].

**Table 1:** State-of-the-art implementations of nGVS for VH management.

Year (Ref.)	Experiment	Device Description	Outcome
**2024 ([79])**	A total of 16 patients with progressive supranuclear palsy were administered nGVS.	nGVS was administered bilaterally, using an amplitude of 0 to 0.7 mA, a frequency range of 0 to 30 Hz, and some additive noise.	Nine out of sixteen patients showed improvements in balance and reduction in body sway, which demonstrates that nGVS has utility beyond VH.
**2024 ([80])**	A total of 19 patients with BVH were administered nGVS.	nGVS was administered bilaterally, using an amplitude of 0 to 0.7 mA, a frequency range of 0 to 30 Hz, and some additive noise.	More than half of the assessed patients showed robust improvements in postural balance when treated with nGVS.
**2023 ([81])**	A total of 20 healthy subjects were administered with nGVS, and their center of pressure sway path length was measured.	nGVS was administered bilaterally, using an amplitude of 0.2 mA and 0 mA for normal and sham stimulation, respectively, a frequency range of 0.1 to 640 Hz, and some additive noise.	nGVS demonstrated potential for improving the standing balance function.
**2023 ([9])**	In 11 patients with BVH, optimal balance stabilization was achieved using nGVS.	nGVS was administered bilaterally, using an amplitude of 0 to 0.7 mA, a frequency range of 0 to 30 Hz, and some additive noise.	Improvements in the body’s balance was due to enhancements in vestibular perception.
**2022 ([57])**	nGVS timing was synchronized with the visual motion for 47 participants using a VR head-mounted display.	Used a VR head-mounted display to present real-world 360° videos from a moving first-person viewpoint. nGVS were specifically tuned to match the motion displayed in the videos.	GVS significantly lessened discomfort for VR users prone to cybersickness, enhancing their immersive experience.
**2022 ([66])**	A total of 12 out of 23 patients with BVH received nGVS	nGVS was administered bilaterally, using an amplitude of 0 to 0.7 mA, a frequency range of 0 to 30 Hz, and some additive noise.	BVH patients’ balance improved immediately with nGVS. However, nGVS and VRT did not show synergistic effects, suggesting they might be complementary therapies.
**2021 ([82])**	The standing stability of subjects administered with nGVS was investigated, consisting of 10 BVH patients and 16 healthy patients.	nGVS was applied bilaterally. The stimulation had amplitudes of 0–1 mA and a frequency range of 0.02–10 Hz with additive noise.	nGVS significantly reduced swaying in both healthy individuals and BVH patients, particularly under visually deprived conditions.
**2020 ([64])**	The center of pressure and muscle activity for 17 healthy patients were tested with and without nGVS.	nGVS was administered bilaterally, using an amplitude of 1 mA and 0 mA for normal and sham stimulation, respectively, with a stimulation time of 40 s.	nGVS enhanced balance and muscle activity in the legs, regardless of whether the eyes were closed or open.
**2019 ([83])**	A total of 30 patients were administered posture-improving nGVS.	nGVS was applied bilaterally. The stimulation had amplitudes of 0–1 mA and a frequency range of 0.02–10 Hz with additive noise.	nGVS improved posture in balance-impaired subjects and enhanced natural balance mechanisms, even in those balancing with closed eyes.
**2018 ([84])**	Comparison between three nGVS experiments involving 18, 24, and 16 patients using sham (0 mA), 0.4 mA, and 1.0 mA stimulation, respectively.	nGVS (0.1–640 Hz) was delivered bilaterally at 0.4 and 1.0 mA.	nGVS used cathodal and anodal currents to enhance balance in daily activities like standing, with lasting benefits post-use. This was measured using the center of pressure sway.
**2018 ([65])**	Postural movements were monitored for 13 BVH patients that received nGVS.	nGVS was applied bilaterally. The stimulation had amplitudes of 0–1 mA and a frequency range of 0.02–10 Hz with additive noise.	nGVS offered immediate stability and balance in BVH patients, with effects lasting several hours post-stimulation.
**2017 ([85])**	A VR wearer controlled in a room using nGVS.	GVS induced “galvanic body sway” in standing users and impacts balance in walking users, causing them to stagger in anodal direction. In GalVR, GVS is used for smooth turning during walking.	The study demonstrated GVS’s ability to smoothly divert a user’s planned path in a VR environment (i.e., as a navigation interface).
**2016 ([86])**	nGVS was administered to VH patients.	nGVS was applied bilaterally.	nGVS led to lasting postural stability improvements, persisting even after the stimulus was discontinued.
**2015 ([87])**	Three nGVS experiments were conducted involving six, eight, and five participants, respectively.	The roll, pitch, and yaw motion of the head were dictated by current paths created by GVS administered between the two mastoids and the forehead.	The study demonstrated the potential for directionally induced head motion using GVS.

**Table 2:** Summary of common vestibular prostheses (modified from [77]).

Vestibular Implant (VI)	Sensor	Stimulation Profile	Electrode Placement	Outcome
**VI UW/Nucleus freedom**	None	Fixed pulse rate.	Inserted at the perilymphatic space adjacent to the ampulla of the three semicircular canals.	Modulation of eye movements.
**VI Geneva-Maastricht**	3D gyroscope	Stimulation amplitude and frequency linearly modulated using movement.	Inserted in the ampulla of the three semicircular canals.	Restoration of VOR, VCR, VSR, and cognitive response in patients.
**LD-MVI**	3D gyroscope	Used a 32-point piecewise-linear sigmoid curve to map motion to the stimulation profile (amplitude and frequency).	Inserted in the three semicircular canals ampullae.	VOR restoration, gait, position, and stabilization. Significant improvement in the quality of life.
**Bionic vest**	None	Fixed pulse rate and amplitude.	Inserted adjacent to the saccular nerve.	Gait and gaze stabilization, with improvements in the quality of life.

Artificial hair cells are intended for sensory substitution like vestibular implants; however, instead of using an array of electrodes, they employ synthetic hair cells that interface with excitable neurons of the vestibular system [71]. Biomimetic hair cells are intended to provide better electrical encoding compared to vestibular implants. However, it is still unclear how the artificial hair cells will integrate with the vestibular system [71]. For seamless integration of the artificial hair cells into the inner ear, they have to be small enough to fit into the existing cellular configuration of lesioned hair cells. Also, they need to be biocompatible such that they connect well to the sensory neurons. Due to the synaptic space between the hair cells and sensory neurons, there is no direct connection. As such, some form of “wireless coupling” between the artificial hair cells and the sensory neuron needs to be created. Some of these requirements make the integration of the artificial hair cell with the vestibular system extremely challenging.

## 5. The Future of Wearable Technologies for VH Management

Wearable devices enhance healthcare outcomes by enabling continuous monitoring and providing objective measurements, which are crucial for managing VH. These devices can improve quality of life by reducing dizziness, enhancing balance, and decreasing fall risks. They also facilitate accurate diagnosis and personalized treatment plans by tracking symptoms precisely. The following sections will detail the essential requirements for VH management, which could help us identify areas where current solutions may be lacking, areas where improvements or innovations are needed, and solutions that directly address the needs of patients.

### 5.1. Essential Requirements for Wearable Systems for Vestibular Management

Wearable technology offers many benefits in vestibular management and is an active research field for academic and industrial communities. However, current wearable technologies are mostly consumer-oriented gadgets and might not be suitable for detailed medical monitoring or diagnosis. For a wearable system to be of clinical value in an ambulatory and home environment, wearable devices need to meet the following essential requirements.

#### 5.1.1. Objective and Comprehensive Data Collection

Traditional patient assessments often rely on methods like questionnaires and subjective tests, which can be a tedious process and inaccurate [88]. For proper VH management, objective and comprehensive data that accurately reflect patients’ functional ability in their daily environment are required. This can be achieved using wearable sensors that capture objective physiological data such as head and eye movement, body sway, posture, and gait. 

#### 5.1.2. Advanced Data Analysis

Effective analysis of sensor data can lead to tailored solutions to improve the quality of healthcare. This prepares healthcare providers for appropriate action and enables more personalized treatment plans. Wearable systems, with advanced data analysis capabilities, can extract meaningful insights from the vast data they collect [88]. These insights, interpreted through machine learning and other sophisticated algorithms, can predict symptom onset and identify significant anomalies for understanding VH [89]. Studies using data from registries like DizzyReg have successfully differentiated various vestibular disorders using machine learning models [90].

The application of sophisticated algorithms and techniques can yield the same results with less data, or when using the same amount of data, it can provide a more precise outcome, better differentiation among variables, and quicker conclusions. For proper VH management, wearable devices with advanced data handling capabilities provide a unique opportunity to understand user behavior, predict future needs, and make informed decisions about vestibular management.

#### 5.1.3. Real-Time Monitoring and Prediction

To mitigate dizziness, improve balance, and lower the risk of falls, techniques that can quickly respond after sensing are crucial. These techniques should be able to make predictions with minimal time lag and generate meaningful output to guide treatment strategies. Wearable devices that provide real-time feedback on a patient’s condition can fulfill this need, enabling quick adjustments to therapy or disease prediction [88]. These devices monitor VH symptoms in real-time using various feedback signals, including bioelectrical signals like ENG, EEG, and EMG for eye-movement tracking and biophysical signals for head and body movement measurements. Some devices, such as the one used in a recent study on real-time postural balance and gait training, use a closed-loop system with vibrotactile feedback [91]. This system quickly senses posture deviations, provides corrective vibrotactile feedback, and ensures effective rehabilitation and proper posture training, highlighting the importance of quick response after accurate sensing.

### 5.2. Future Research Directions for Vestibular Management Wearables

**Figure 5:**
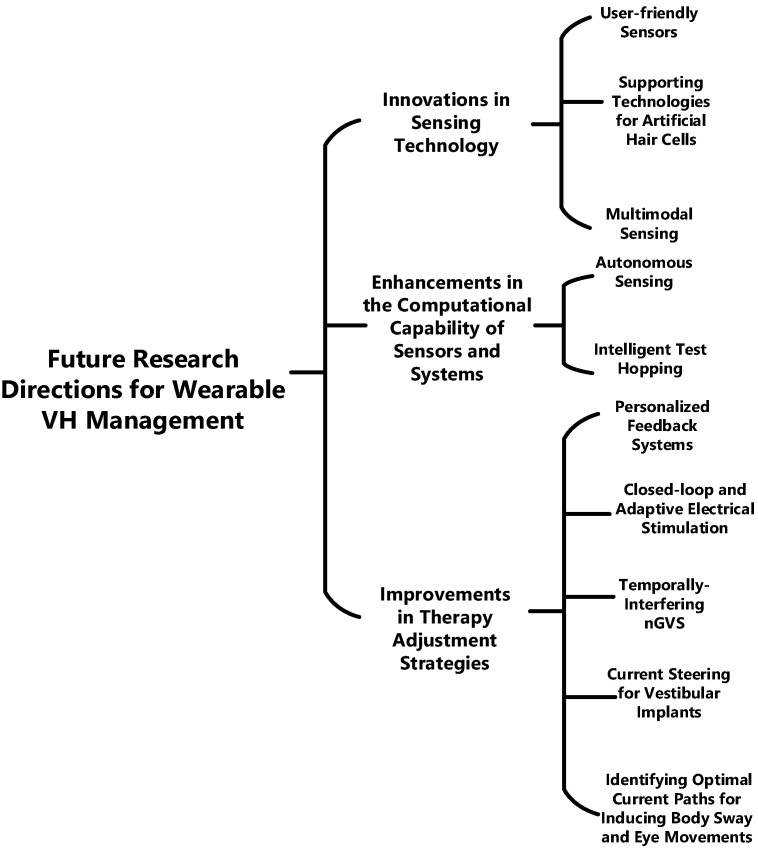
Summarizing Future Research Directions for Wearable VH Management.

In addition to the standard low-power and lightweight requirements for generic wearable sensors, wearables for VH have several aspects worth further research and exploration. Figure 5 highlights key areas that could transform VH management, with subsequent sections offering more in-depth information.

#### 5.2.1. Innovations in Sensing Technology

The vestibular system detects and controls body, head, and eye movements in three-dimensional space by integrative action of semicircular canals, otolith, visual, somatosensory, and motor activity [92]. This results in a pervasive need for sensing and computing capabilities in order to enable proper management of stability, sway, gait, and gaze of patients. In addition, since the VOR continues to function even when the eyes are closed, methods that measure eye movement when the eyes are closed may also be required. Future wearable systems are expected to feature enhanced sensor technology for more accurate and reliable data collection [2]. This could lead to better diagnosis and treatment of VH. This paves the way for new possibilities in sensor technology, outlined as follows:

**(A)**  **User-friendly Sensors:** For proper VH management using wearables, it is very likely that devices will be worn by users for an extended period of time. Thus, user-friendly sensors are crucial for VH management. This makes factors like comfort, size, weight, and power essential for these devices. For instance, eye tracking, which is a key component of all clinical tests for VH, is predominantly used in these wearables. However, traditional eye-tracking methods may block vision and interfere with normal daily routines, posing a significant challenge. Therefore, it is essential to explore innovative technologies that can measure eye movements indirectly or track alternative indicators representative of eye movements. These sensors, combined with miniaturized, contactless readout electronics, require less energy, extending the device’s battery life. Such advancements would not only enhance user comfort but also ensure that wearables seamlessly integrate into users’ lives without disrupting their vision and daily activities. This approach would significantly improve the usability and acceptance of these wearables, thereby facilitating more effective management of VH.
**(B)**  **Multimodal Sensing:** Since VH is a result of the mismatch between the head and some components of the nervous system, sensors in multiple locations are required for proper VH monitoring. This leads to a more precise prediction of dizziness onset, risk of fall, and balance instability. In order to facilitate multimodal sensing, data from the multiple sensors needs to be integrated. Aside from multimodal sensing, even if a single feature like posture is being measured in a patient, say, using an IMU. The dynamic state of a subject affects the IMU sensitivity. This means that the IMU’s sensitivity and accuracy can vary depending on the subject’s activity [93]. Sensor fusion, consisting of multiple sensors, can be used to overcome the limitations of using a single sensor.
**(C)**  **Supportive Technologies for Seamless Integration of Artificial Hair Cells with the Inner Ear:** Even though studies like [8] have developed biomimetic hair cells capable of detecting low-frequency movements with high sensitivity, the challenge still remains integrating these sensors into the extracellular environment of the inner ear. This opens up several research opportunities on supportive technologies that could assist with the seamless integration of artificial hair cells with the inner ear; below are some potential areas:
  - **Material Technology:** This could involve the development of biocompatible materials that can better interface with biological tissues of the inner ear without resulting in implant rejection or causing damage/inflammation.
  - **Nanotechnology:** This could involve creating artificial hair cells that are nearly the same size and shape as the natural hair cells of the inner ear. This would ensure the seamless integration of the hair cells into the existing cellular configuration.
  - **MEMS Transducers for Sensorineural Coupling:** Designing highly efficient MEMS interfaces that can effectively transmit signals from the artificial hair cells to the vestibulocochlear nerve. This could involve the development of novel microelectrode arrays or MEMS transducers that enable coupling between the artificial sensors and the nerve.

#### 5.2.2. Enhancements in the Computational Capability of Sensors and Systems

Below are some ways the computational capability of wearable systems for VH can be enhanced:

**(A)**  **Autonomous Sensing:** VH is predominant among the elderly, who may be less inclined to frequently calibrate or recalibrate wearable devices. Making the need for more intelligent sensors that can process and interpret data at the point of collection essential. Autonomous sensors can fulfill this role in VH management by detecting abnormal patterns in a subject’s gait or eye movements, facilitating the tracking of disease progression. These sensors can be adaptive, adjusting the threshold for detecting abnormalities based on the patient’s condition. This adaptability is particularly useful when real-time therapy is administered. For instance, during the early stages of VH, when symptoms are more manageable, high precision may be required to provide therapy only when necessary, thereby minimizing side effects. Conversely, in advanced stages of VH, a higher sensitivity may be needed to identify most manifestations as the condition becomes less manageable. Also, autonomous sensors have limited bandwidth and power requirements since they make decisions onsite without the need to transmit them to another device.
**(B)**  **Intelligent Test Hopping:** Currently, clinics are using VH screening tests selectively, which may not offer a holistic assessment. The feasibility of conducting comprehensive tests each time is often limited by time and cost constraints. To circumvent this, the creation of heuristic-based algorithms or systems embodying the concept of intelligent test hopping could streamline the evaluation process. These systems, designed as wearable devices for ease of use and continuous monitoring, could displace the current series of narrow bandwidth tests. Intelligent test hopping allows for the selection of the most relevant tests based on real-time data, thereby optimizing the assessment process. This paves the way for home-based VH assessments or tests.

#### 5.2.3. Improvements in Therapy Adjustment Strategies

Developments in AI and machine learning could equip future wearable systems with advanced data analysis capabilities [2], leading to insightful data interpretations and improved patient management. This opens up new opportunities in patient-specific therapy, which are summarized below:

**(A)**  **Personalized Feedback Systems:** Future systems for VH management could offer patient-specific therapy, providing feedback (visual, auditory, or haptic) based on patient status. This necessitates research on innovative feedback systems. Examples of these include the following:
  - **Dizziness Alerting System:** Personalized for patients with dizziness, a major symptom of VH. An example is the Continuous Ambulatory Vestibular Assessment, which detects dizziness by recording head and eye movements [94]. However, it lacks a patient alerting mechanism, which could use auditory or haptic feedback.
  - **Balance Improvement using Sensory Feedback:** Unsteadiness, a common VH symptom, can be managed using feedback like vibrotactile feedback. An example is a vibrotactile belt used to improve the perception of verticality in patients during daily activities [44]. These devices should be comfortable and not provide unwanted sensations.
**(B)**  **Closed-loop and Adaptive Electrical Stimulation:** Understanding the main causes of vestibulopathy is crucial for effective management [71]. This paves the way for personalized treatment plans. One such approach is the use of closed-loop and adaptive electrical stimulation. In these systems, the output is continuously monitored, and the input is adjusted based on deviations from a predetermined baseline. This strategy, employed in VH management through an amplitude-modulated signal from a vestibular prosthesis, holds promise for effective therapy adjustment. However, the optimal approach for closing the loop, be it sensory augmentation or sensory substitution, remains to be determined. The effectiveness of these systems hinges on advancements in sensing technology, feedback systems, and data processing algorithms.
**(C)**  **Identifying Optimal Current Paths for Inducing Body Sway and Eye Movements:** Studies have demonstrated the existence of current paths between the left and right mastoids, as well as the left and right forehead, for inducing body sway or eye movements [57,87]. Noisy GVS has the capacity to induce eye movements without any head or body motion [63] and generates body sway during standing or walking. This dual influence makes it a valuable tool for diagnosing and managing VH. For triggering complex trajectories in eye movement and body sway, it is essential to establish the optimal current path for inducing these effects. This could involve identifying the optimal number of electrodes based on the intended eye movement, the optimal position for the placement and arrangement of these electrodes, and the way electrical currents are distributed among the electrodes.
**(D)**  **Focused and Targeted Electrical Stimulation:** There is a need for motion-modulated electrical stimulation in VH treatment, achievable through vestibular implants or non-invasive nGVS systems. This involves controlling the electrical stimulation that is delivered to the vestibular system. Given the inherent risks of invasive inner ear surgery with implants, nGVS may be a preferable alternative [30]. To target the semi-circular canal more precisely using nGVS, sophisticated algorithms, such as AI-based ones, or adaptive stimulation profiles could be used for beamforming or temporally interfering (TI) stimulation, minimizing side effects from electric current spread to nearby organs. TI uses a low-frequency envelope waveform generated by the superposition of two high-frequency sinusoidal currents of slightly different frequencies to stimulate specific targets inside the brain [95]. This method holds the advantages of both spatial targeting and non-invasive stimulation and could also be adopted for the vestibular system. For vestibular implants, current steering for improved spatial precision can be adopted.

## 6. Conclusions

Wearable technologies for VH management are mainly required at the advanced stages of VH, when VRT may not be effective. The aim of wearable technologies for VH management is to restore or replace vestibular function. This can be achieved through sensory substitution or sensory augmentation. Using sensory augmentation, biofeedback devices can be designed that provide patient-specific management based on the feedback signals patients are most attuned with—visual, auditory, or vibrotactile. However, it is still not clear if this could be adopted for long-term use. For long-term use, nGVS may be a better alternative than biofeedback sensory augmentation. Nonetheless, some challenges may need to be addressed to improve the utility of nGVS, which include improving stimulation focus, identifying the optimal current paths that induce body sway and eye movements, determining the optimal stimulation parameters, establishing the optimal amount of noise that may be required for each symptom, as well as how long potential benefits may last. In the case of completely lost vestibular function, sensory substitution may be more appropriate. Using vestibular implant-based sensory substitution, future work needs to optimize the electrical stimulation patterns such that they improve the dynamic range, the selectivity, and the vestibular reflexes. Also, more futuristic solutions like biomimetic hair cells intend to provide better electrical encoding for sensory signals compared to vestibular implants. However, it is still unclear how the artificial hair cells will integrate with the vestibular system. In the coming years, the key challenges that may need to be solved to advance wearable solutions for VH management include innovations in sensing technology, enhancements in the computational capability of sensors and systems, improvements in therapy adjustment strategies, and targeted and less invasive neuromodulation.

## Abbreviations

Bilateral Vestibular Hypofunction	BVH
Galvanic Vestibular Stimulation	GVS
Inertial Measurement Unit	IMU
Noisy Galvanic Vestibular Stimulation	nGVS
Stochastic Resonance	SR
Unilateral Vestibular Hypofunction	UVH
Vestibular Hypofunction	VH
Vestibular Implant	VI
Vestibular Rehabilitation Therapy	VRT
Vestibulocollic Reflex	VCR
Vestibulo-ocular Reflex	VOR
Vestibulo-spinal Reflex	VSR
Virtual Reality	VR
